# Diagnostic Accuracy of Dermoscopy of Actinic Keratosis: A Systematic Review

**DOI:** 10.5826/dpc.1004a121

**Published:** 2020-10-26

**Authors:** Karla L. Valdés-Morales, María Luisa Peralta-Pedrero, Fermín Jurado-Santa Cruz, Martha Alejandra Morales-Sánchez

**Affiliations:** 1 Centro Dermatológico Dr. Ladislao de la Pascua, Mexico City, Mexico

**Keywords:** actinic keratosis, dermoscopy, dermatoscopy, diagnostic accuracy

## Abstract

**Introduction:**

Dermoscopy is a tool that aids clinicians in the diagnosis of actinic keratosis; however, few diagnostic accuracy studies have determined its sensitivity and specificity for this diagnosis.

**Objective:**

Determine the diagnostic accuracy of dermoscopy on actinic keratosis.

**Methods:**

A systematic review was conducted on EMBASE, PubMed, Scopus and the Cochrane Central Registry of Controlled Trials from inception to August 2019.

**Results:**

We screened 485 titles and abstracts. Two studies comprising 219 actinic keratoses were eligible for qualitative analysis. The number and heterogeneity of included studies limited a quantitative analysis.

**Conclusions:**

Studies that focus specifically on the diagnostic accuracy of dermoscopy for actinic keratosis are lacking.

## Introduction

Actinic keratosis (AK) is a cutaneous neoplasm that arises on chronically sun-exposed skin. For years, AK was considered to be a separate entity from squamous cell carcinoma (SCC) [1], a premalignant lesion; however, in recent years this concept has been challenged, and now most authors consider AK the continuum of SCC [2–4]. The rate of progression to SCC differs greatly among the studies, ranging from 0.025%–20% per year [5,6]; however, it has been documented that 60%–80% of SCCs arise from AK. Correct diagnosis is important for prompt treatment [5].

Dermoscopy is a tool that aids in the clinical diagnosis of multiple melanocytic and non-melanocytic lesions. Several dermoscopic patterns for the detection of AK have been described: gray structures, scale, and rhomboidal lines, among others, for pigmented AK [7–9], and linear wavy vessels, follicular plugs surrounded by a pink-red pseudonetwork, and scaling have been described in nonpigmented AK [10]. Despite the widespread use of dermoscopy, there are no recent systematic reviews that report the sensitivity and specificity of this tool in the diagnosis of AK.

## Methods

We conducted a systematic review of the literature in accordance with the Preferred Reporting Items for a Systematic Review and Meta-analysis of Diagnostic Test Accuracy Studies (PRISMA-DTA Statement) [11] and the Cochrane Handbook for Systematic Reviews of Diagnostic Test Accuracy [12]. This protocol was registered on the International Prospective Register of Systematic Reviews (CRD42019116000).

The main purpose of this study was to determine the sensitivity and specificity of dermoscopy for the diagnosis of AK.

### Inclusion/Exclusion Criteria

We included studies in which participants were adults (>18 years old) and studies that followed a diagnostic accuracy study flow, ie, patients with suspicion of AK underwent a dermoscopic examination (index test) then a histopathological study (reference standard test). Published articles written in the English or Spanish language that followed this study flow were included in the systematic review. Studies in which participants had the histopathological diagnosis of AK prior to examination of dermoscopic images by an evaluator, studies in which the clinical and dermoscopic diagnosis was not blinded from a dermatopathologist, and case reports were excluded.

### Data Extraction and Analysis

A literature search was conducted on EMBASE, MEDLINE, Scopus, and the Cochrane Central Registry of Controlled Trials from inception to August 2019. The key words used were “actinic keratosis” and “dermoscopy,” as well as its synonyms “dermatoscopy” and “epiluminescence microscopy.” Studies that met the criteria were retrieved and reviewed by 2 researchers, and discrepancies were settled by a third researcher. The extracted information included study type, number of patients in each study, patient characteristics, type of test, and reference standard. Two authors independently extracted these data, and discrepancies were identified and resolved by discussion with a third reviewer.

Applicability and risk of bias was assessed using the QUADAS-2 instrument [13], including every checkpoint except the appropriate interval between index test and reference standard test, which was not applicable to this clinical scenario. The reference standard test was the histopathologic study.

## Results

**Figure 1:**
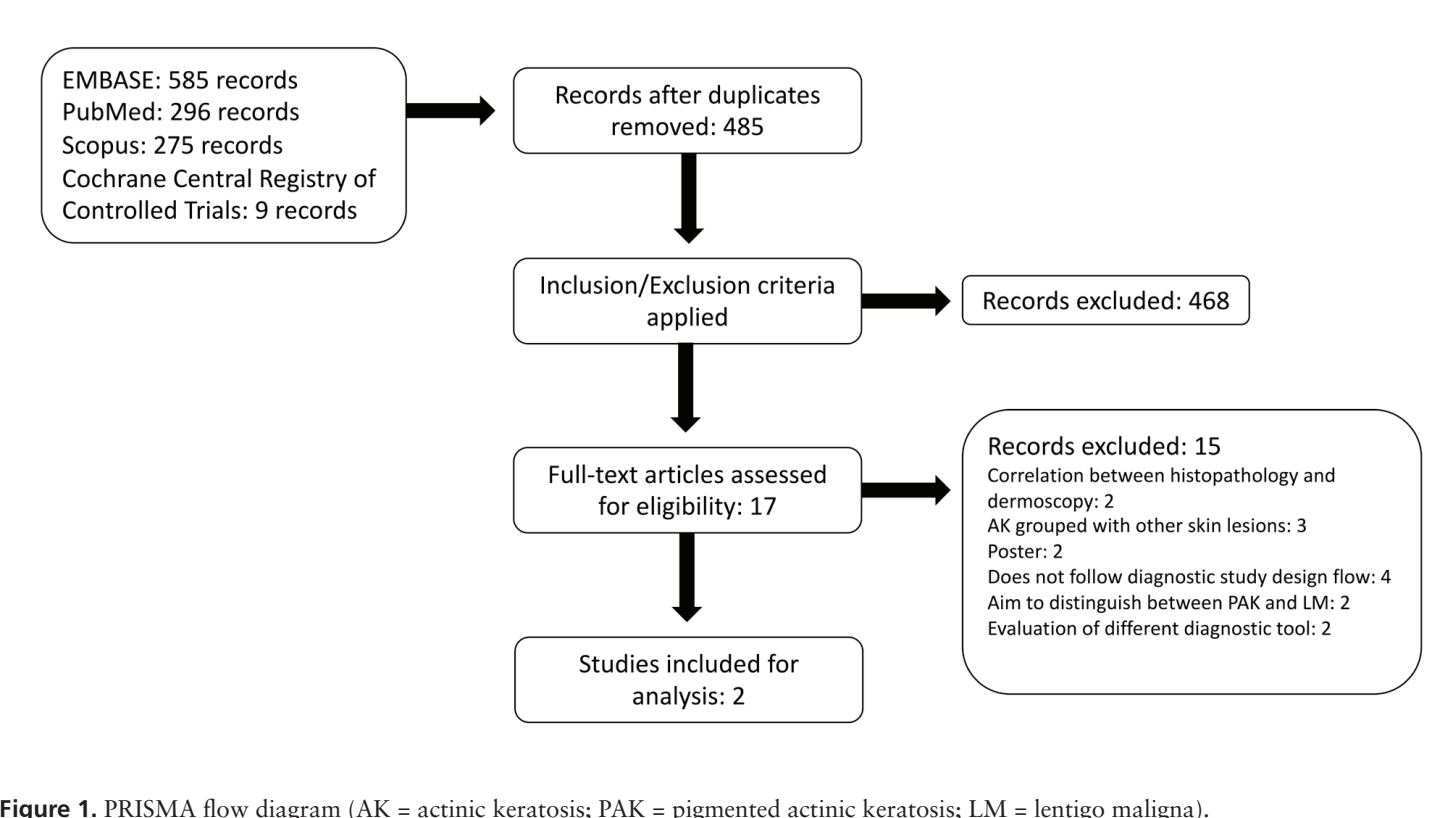
PRISMA flow diagram (AK = actinic keratosis; PAK = pigmented actinic keratosis; LM = lentigo maligna).

The search yielded a total of 1,165 studies; duplicates were removed, and a total of 485 titles and abstracts were reviewed (Figure 1). Seventeen studies were read in full text, and 2 of them fulfilled eligibility criteria, with a total of 219 actinic keratoses in 210 patients (Table 1). Both studies showed a male predominance, as well as a mean age that ranged between 67 and 69 years. The studies were held in Australia, Italy, USA [14], and Spain [15].

Of the 15 excluded studies, exclusions were mainly due to study flow and to different study objectives, for example, determining correlation between histopathology and dermoscopy [16,17], aiming to distinguish between pigmented AK and lentigo maligna [7,18], determining dermoscopic pattern frequency [19], or evaluation of a different diagnostic tool [20,21], among others. Excluded studies and reasons for exclusion are presented on Supplementary Table 1.

The QUADAS-2 risk of bias and applicability assessment is shown in Table 2. The reference standard and index test had a low risk of bias in both studies [14,15], however, the flow and timing as well as patient selection had a high risk of bias in the Zalaudek et al [14] study, as the study design was not clearly stated. On the other hand, both studies show a low concern regarding applicability.

Both studies evaluated the characteristics of dermoscopic photographs, including erythematous pseudonetwork, surface scale, linear wavy vessels, and follicular plugs. Zalaudek et al added coiled and dotted vessels to the dermoscopic features being evaluated. Sensitivity and specificity of dermoscopy for the diagnosis of AKs were calculated by Huerta-Brogeras et al [15] with a sensitivity of 98.7% and specificity of 95%. In the second study [14], all but one dermoscopist suspected AK as the initial diagnosis with an overall sensitivity of 97.5%; however, in 19 of those lesions, the initial diagnosis also included Bowen disease or superficial basal cell carcinoma. If we consider these cases as negative tests, the calculated sensitivity would decrease to 51.2%.

Overall, the most common dermoscopic finding was surface scale (86.7%), followed by follicular openings (83.1%) and erythematous pseudonetwork (79.9%), both comprising the “strawberry pattern,” and lastly, linear wavy vessels (71.2%). Important clinical and methodological heterogeneity between the studies was considered, so a pooled sensitivity and specificity was not calculated.

## Discussion

In this review, 2 studies fulfilled inclusion criteria with a low risk of applicability; Huerta-Brogeras et al had a low risk of bias, whereas Zalaudek et al had high risk of bias, mainly due to study flow and timing, not clearly specifying eligibility and exclusion criteria, as well as timing of the histopathological diagnosis within the study flow. The most common dermoscopic finding in both studies was the presence of a red pseudonetwork surrounding follicular openings comprising the “strawberry pattern” [14].

Despite the widespread use of dermoscopy for AK, few studies that prospectively evaluate its sensitivity and specificity have been published. Descriptive studies have been completed, wherein the frequency of each dermoscopic sign [19] or its correlation with histopathological findings are reported [17,22]. Of 70 AKs studied by Zalaudek et al in 2012, the red pseudonetwork was the most frequent finding (67.1%), followed by scales and targetoid hair follicles [23]. Kelati et al described dermoscopic findings in 232 cases of facial pigmented AK, and the most frequent findings were: rhomboidal appearance (82.8%), inner gray halo (58.6%), scales (39.2%), jelly sign and superficial pigmentation (37.5%), among others [19]. Lee et al examined the correlation between dermoscopic and histopathological findings in Korean patients. Among the results was the frequency of dermoscopic findings in 61 AKs from 47 patients: white yellow scale (73.1%), targetoid sign (65.4%), white structure-less area (50%), red background (50%), red pseudonetwork (46.2%), and rosettes (7.7%) [17].

Studies comparing dermoscopic features to distinguish between lentigo maligna and pigmented AK have been published [7,9,18]. Akay et al evaluated dermoscopic parameters of lentigo maligna in facial pigmented skin lesions; 67 pigmented AKs were included in the study, and the frequency of dermoscopic findings was reported: slate gray dots (70%), annular-granular pattern (39%), brown-to-gray pseudonetwork (36%), and rhomboidal structures (36%) were the most frequent. In their study, the presence of pseudonetwork was found to be specific for pigmented AK [18].

In 2012, Rosendahl et al [24] conducted a diagnostic accuracy study of dermoscopy on melanocytic and non-melanocytic pigmented lesions, and a few years later, Gomez-Martin et al [25] published a diagnostic accuracy study of dermoscopy and reflectance confocal microscopy on pink flat lesions of the legs. Both studies followed an adequate diagnostic accuracy flow design and performed a quantitative analysis of data, including sensitivity and specificity; however, even though AKs were included in both studies, Gomez-Martin et al grouped AKs with SCC and Bowen disease, while Rosendahl et al considered AK a superficial variant of SCC and grouped them with malignant lesions in the study. Bowen disease and SCC are the main differential diagnoses of AK, and the lack of specific results for AK was the reason for exclusion of these studies from the present review.

The most significant limitation of this study is the inclusion of only 2 studies in our analysis. The aim of this study was to perform a diagnostic accuracy systematic review with a meta-analysis; to achieve this, we only included studies in which the study flow of a diagnostic accuracy study was followed (clinical suspicion followed by performance of index test followed by the reference standard test) in order to calculate sensitivity and specificity. The initial study was designed to include only nonpigmented AK, then we broadened our inclusion criteria to both pigmented and nonpigmented AK. However, after a thorough review of the literature, 17 studies evaluated dermoscopy as a diagnostic tool for AK, and only 2 studies followed this flow. Most of the studies that evaluate dermoscopic characteristics follow a different study flow: dermoscopic findings are retrieved from dermoscopic images with known histopathological diagnosis. This low number of included studies led to the impossibility of performing a quantitative analysis of data.

Multiple lesions on chronically photodamaged skin can lead to the clinical diagnosis of AK; a single AK, on the other hand, may present a more difficult clinical scenario in which dermoscopy plays a determining role in diagnosis. The characteristic “strawberry pattern” is most frequent in facial lesions but is not commonly found in extra-facial regions [26,27]. This characteristic pattern may also be absent in different types of AK, such as bowenoid AK, where a vascular pattern of glomerular or coiled vessels may be seen [26], or hyperkeratotic AK, where scale is the predominant feature. Future studies that examine the dermoscopic pattern of AK on non-facial topographies, as well as of different types of AKs, are needed.

Huerta-Brogeras et al [15] excluded lesions that upon clinical examination were suspected to be malignant. In future diagnostic accuracy studies, malignant and equivocal lesions should be included because the clinical differential diagnosis of AK includes Bowen disease, invasive SCC, superficial basal cell carcinoma, and even granulomatous and inflammatory conditions [28–30]. Lesions that may resemble AK clinically should be included in diagnostic accuracy studies and subjected to the dermoscopic and histopathological examinations to objectively measure precision of dermoscopy. Guidelines for reporting diagnostic accuracy studies (STARD) were updated on 2015, recommending key points for the elaboration and publication of these types of studies [31]; this allows for more homogeneous study designs to be accomplished in diagnostic accuracy studies.

Throughout the literature review, we noted different dermoscopic terms for similar dermoscopic structures and patterns among studies. For more uniform language, dermatologists should adhere to the standardized dermoscopic terminology published by the International Dermoscopy Society [32,33] both for academic and clinical studies. Having uniform dermoscopic terminology will enable more homogenous and comparable studies and will facilitate dermoscopic training.

## Conclusions

Dermoscopy is a practical tool to aid in the diagnosis of AK; however, studies that focus specifically on the diagnostic accuracy of dermoscopy for actinic keratosis are lacking.

## Supplementary Information

**Supplementary Table 1:** Excluded Studies With Reason For Exclusion

Authors	Title	Reason for Exclusion
Lee et al [ 1]	Correlations between histopathologic and dermoscopic findings in Korean actinic keratosis.	Correlation between histopathology and dermoscopy. Retrospective study of nonpigmented AKs; aimed to describe histopathological findings with dermoscopic ones. Description of dermoscopic features’ frequency.
Micantonio et al [ 2]	A new dermoscopic algorithm for the differential diagnosis of facial lentigo maligna and pigmented actinic keratosis.	Aimed to distinguish between PAK and LM. Dermoscopic patterns to distinguish PAK from LM.
Gómez-Martín et al [ 3]	Diagnostic accuracy of non-melanocytic pink flat skin lesions on the legs: Dermoscopic and reflectance confocal microscopy evaluation.	AK grouped with other skin lesions. Study included all pink lesions. The clinical suspicion is divided into 2 groups: malignant or benign, does not give data specifically of AK (AK grouped with inflammatory disease group).
Kelati et al [ 4]	Dermoscopy of pigmented actinic keratosis of the face: a study of 232 cases.	Does not follow diagnostic study design flow. Determines frequency of dermoscopic signs.
Lee et al [ 5]	Correlations between dermoscopic and histopathologic findings in actinic keratosis.	Poster. Article from this poster was published in 2019 1.
Li and Chang [ 6]	The investigation of dermoscopy in differential diagnosis of facial actinic keratosis.	Poster.
Lallas et al [ 7]	Dermoscopic clues to differentiate facial lentigo maligna from pigmented actinic keratosis.	Does not follow diagnostic study design flow. Aim of the study was to determine the frequency of the dermoscopic criteria for facial pigmented lesions.
Elwan et al [ 8]	Dermoscopic and histopathological correlation in some epidermal tumors: A preliminary study.	Does not follow diagnostic study design flow. The study aimed to study epidermal tumors (BCC, SK, AK, and SCC).
Tschandl et al [ 9]	Dermatoscopy of flat pigmented facial lesions.	AK grouped with other skin lesions. Considers PAK and pigmented Bowen’s disease as one group.
Rosendahl [ 10]	Diagnostic accuracy of dermatoscopy for melanocytic and nonmelanocytic pigmented lesions.	AK grouped with other skin lesions. Adequate study design and flow, however, it groups all skin lesions into 2 groups: malignant or benign. No specific data on AKs.
Akay et al [ 11]	Dermatoscopy of flat pigmented facial lesions: Diagnostic challenge between pigmented actinic keratosis and lentigo maligna.	Aimed to distinguish between PAK and LM. Lesions were included only if they presented with specific patterns of LM.
Cinotti et al [ 12]	Dermoscopy vs. reflectance confocal microscopy for the diagnosis of lentigo maligna.	Evaluation of different diagnostic tool. Diagnostic accuracy study of dermoscopy and RCM for the diagnosis of LM.
Wurm et al [ 13]	The value of reflectance confocal microscopy in diagnosis of flat pigmented facial lesions: a prospective study.	Evaluation of different diagnostic tool. Aim of the study was to describe utility of confocal microscopy on different flat, pigmented lesions.
Guitera et al [ 14]	Dermoscopy and in vivo confocal microscopy are complementary techniques for diagnosis of difficult amelanotic and light-coloured skin lesions.	Does not follow diagnostic study design flow. Study included different amelanotic and light-colored lesions. Aim was for diagnosis of melanoma.
Stoica et al [ 15]	Dermatoscopic and histopathological aspect of preneoplasia and skin cancers - study on 74 patients.	Correlation between histopathology and dermoscopy. Aimed to correlate the dermoscopic and histopathological aspect of tumors.

AK = Actinic keratosis; PAK = pigmented actinic keratosis; LM = lentigo maligna, RCM = reflectance confocal microscopy

## Figures and Tables

**Table 1:** Study Characteristics of Included Studies

Study Characteristics	Zalaudek, 2006 [14]	Huerta-Brogeras, 2012 [15]
Dermatoscope	Heine Delta 20 hand-held dermatoscope or a Dermlite FOTO lens attached to a Nikon Coolpix 4500 digital camera	Dermlite FOTO lens attached to Canon 400D camera
Included lesions	Nonpigmented AK	Nonpigmented and pigmented AK
Number of patients	32	178
Number of AK	41	178
Age (mean)	69 years	67 years
Clinical examination (naked eye vs. photography?	Photograph	Naked-eye
Dermoscopic examination (real time vs. photography)	Photograph	Photograph
HP study of all lesions	Yes	Yes

AK = actinic keratosis; HP = histopathological study

**Table 2:** Risk Of Bias Assessment And Applicability Using QUADAS-2 Tool

Study (First Author and Year)	Risk of Bias				Applicability		
	Patient Selection	Index Test	Reference Standard Test	Flow and Timing	Patient Selection	Index Test	Reference Standard Test
Huerta-Brogeras, 2012 [15]	Low	Low	Low	Low	Low	Low	Low
Zalaudek, 2006 [14]	High	Low	Low	High	Low	Low	Low
